# Power Amplifier Design for Ultrasound Applications

**DOI:** 10.3390/mi14071342

**Published:** 2023-06-30

**Authors:** Hojong Choi

**Affiliations:** Department of Electronic Engineering, Gachon University, 1342 Seongnam-daero, Sujeong-gu, Seongnam 13120, Republic of Korea; hojongch@gachon.ac.kr; Tel.: +82-31-750-5591

**Keywords:** power amplifier, ultrasound transducer, ultrasound system

## Abstract

A design analysis of the power amplifiers developed for ultrasound applications was conducted because ultrasound applications require different types of power amplifiers, which are one of the most critical electronic components in ultrasound systems. To generate acoustic signals using transducers, which are among the most important mechanical devices in ultrasound systems, an appropriate output voltage, current, or power signal must be produced by a power amplifier. Therefore, an appropriate design analysis of the power amplifier must be conducted to obtain the optimal performance from a transducer. In addition, because of new ultrasound research trends, such as ultrasound systems with other imaging modalities and wireless ultrasound systems, the selection of an appropriate power amplifier could improve the performance of an ultrasound system with other imaging and therapy modalities. This paper describes the design parameters of a power amplifier, including the gain, bandwidth, harmonic distortion, and efficiency. Each power amplifier has specific applications and limitations. Therefore, this review will assist design engineers and ultrasound researchers who need to develop or use power amplifiers in ultrasound applications.

## 1. Introduction

Ultrasound has been widely used in various applications such as imaging, therapy, acoustic stimulation, cell sorting, and neuromodulation [1,2,3,4,5]. Ultrasound systems have been combined with several other modalities, such as magnetic resonance imaging (MRI), optical imaging, and positron emission tomography (PET) [6,7,8]. In addition, advanced integrated circuits have led to the development of wireless ultrasound systems by several companies, including General Electric, Siemens Healthineers, and Philips Medical [9,10,11]. Such an ultrasound system could provide physicians with prompt guidance in ambulances and emergency rooms [12,13].

**Figure 1:**
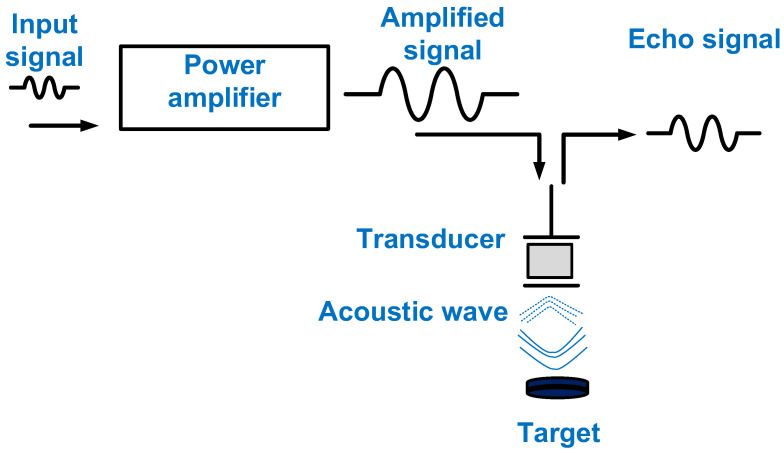
The power amplifier for transducer excitation.

An ultrasound system comprises a transmitter, a transducer, and a receiver [14]. The transmitter and receiver are electronic systems, whereas the transducer is a mechanical device [15]. The transmitter generates a high voltage or current depending on the type of transducer [16,17]. The receiver amplifies the weak echo signal received from the transducer and processes the data to construct images [18]. One of the last-stage electronic components of a transmitter is the power amplifier, which can affect the performance of the transducer [19,20]. Therefore, it is important to consider the performances of power amplifiers. Figure 1 shows the description of the power amplifier for transducer excitation. The input signal is amplified by the power amplifier, and the amplified signal is sent to trigger the transducer, which generates an acoustic wave delivered to the target. Afterward, the echo signal is generated from the transducer.

Basic classifications can be provided for the areas of imaging and therapy in ultrasound research. Imaging is a common technique that uses a transmitter and receiver [21]. Therefore, the power amplifier in a transmitter can affect the performance of the system [22]. In a receiver, the echo signal data received by a transducer are converted into imaging data [23]. For imaging, it is preferable for power amplifiers to have a high gain and wide bandwidth [24]. The power amplifier bandwidth must be at least twice that of the transducer for harmonic imaging [24].

Both high- and low-power therapies are utilized [25]. High-power therapy utilizes high-intensity focused ultrasound (HIFU). The output power from the power amplifier is used to trigger the low-frequency transducer used for HIFU applications, and HIFU is commonly used in conjunction with an MRI machine to monitor sudden temperature variations [26]. For low-power (<1 W/cm^2^) therapy, power amplifiers have also been utilized to trigger the transducer; therefore, only acoustic power or combinational methods using acoustic waves with microbubbles have been used [27,28]. Neuromodulation research can be categorized as low-power therapy. Neuromodulation with acoustic stimulation has recently gained attention because of its ability to deliver drugs to the brain [29]. In neuromodulation research, the power amplifier is the last-stage electronic component before the transducer. The power amplifier needs to have high linearity because researchers do not know the amount of voltage or power that is needed to generate the proper outcome for the desired target [30]. In cell sorting, a transmitter has been used, and optical microscopy has been used to monitor the effect on cells [31]. A power amplifier is used to trigger the transducer to produce variable acoustic power [32,33]. The acoustic power generated is carefully controlled during cell sorting. In a previous study, the amount of output voltage generated from the power amplifier needed to be checked because there were some threshold voltages when producing a sorting effect on the cells [34]. 

Based on its output, a power amplifier can be classified as a voltage- or current-type amplifier, depending on the transducer device [35]. A voltage amplifier is used to generate a voltage output for a transducer that is fabricated using a piezoelectric material [36]. A current amplifier is used to generate a current output for a transducer that is fabricated using a capacitive material. This is known as a capacitive micromachined ultrasonic transducer (CMUT) [37,38]. Such a power amplifier is called a transimpedance amplifier because the input is a voltage, and the output is a current [39].

At the circuit design level, the power amplifiers used for ultrasound applications are classified into linear and nonlinear types based on the conduction angle of the transistor, which is the main component of the power amplifier [40]. Linear power amplifiers include Class-A, -B, and -AB power amplifiers, and nonlinear power amplifiers include Class-C, -D, -E, and -F power amplifiers. For linear power amplifiers, a large transformer-based power supply is required to supply precise direct current (DC) voltages [41]. For imaging applications, Class-A, -B, and -AB power amplifiers have been utilized. Compared with linear power amplifiers, nonlinear power amplifiers might use a switching-mode power supply to reduce the system size [42]. In nonlinear power amplifiers, the conduction angle of the transistor causes different voltage and current phase conditions [43]. Therefore, the power consumption should ideally be minimized. Different design criteria must be considered for each type of power amplifier. For example, in a power amplifier using a feedback resistor, the voltage gain can be reduced to extend the bandwidth, and a nonlinear power amplifier usually has high efficiency but high signal distortion [44]. 

First, database search engines were used to screen the power amplifier articles related to ultrasound research. Duplicate articles were removed from the database search engines. Articles that did not describe the specific parameters of the power amplifiers were excluded. Section 2 describes the design parameters of the different classes of power amplifiers, along with how these are utilized, including the gain, bandwidth, efficiency, and harmonic distortion. Section 3 presents the previously reported power amplifier types for specific ultrasound applications such as imaging, therapy, and power piezoelectric loads. Section 4 presents a discussion and the limitations of the currently developed power amplifiers for ultrasound applications. Finally, Section 5 provides a summary of this study.

## 2. Design Parameters of Power Amplifiers

A transistor is one of the fundamental components of a power amplifier. Therefore, proper transistor selection is the first step in designing a power amplifier [45]. For transistor selection, the circuit designer must examine the specifications of the transistor on the datasheet for discrete components or check the specifications of the transistor in the design kits for the integrated circuit [46,47,48]. The maximum gate–source voltage and drain–source voltage of the transistor must first be checked because the maximum DC bias voltages of the transistor are limited by these voltages [49]. The maximum drain current of the transistor must be considered because the amplified output current level must be lower than the maximum drain current of the transistor [50,51]. The parasitic gate–source, gate–drain, and drain–source capacitances must be considered [52]. The maximum operating frequency of the power amplifier can be limited by the transistor parasitic capacitances [52]. The maximum power consumption must be checked to avoid transistor failure [53]. After the careful selection of the transistor, the design parameters of the power amplifier are considered. The power amplifiers used for ultrasound applications utilize metal-oxide-semiconductor field-effect transistors (MOSFETs) lateral diffusion metal-oxide-semiconductors (LDMOSs), and double-diffused metal-oxide-semiconductors (DMOSs). LDMOSs and DMOSs are types of MOSFETs that operate under high-voltage or high-power conditions [54].

The design parameters must be discussed when selecting appropriate power amplifiers because the power amplifiers must work for transducers and systems. The design parameters of power amplifiers are typically the gain, bandwidth, harmonic distortion, linearity, and efficiency [55,56]. This section guides how to utilize each parameter and the parameters that need to be considered in the power amplifier design. 

The gain of the power amplifier is the extent to which the amplitude of the input signal is amplified to generate the amplitude of the output signal [56]. The bandwidth is an important parameter because the maximum bandwidth of the power amplifier should be higher than that of the transducer. 

Harmonic distortion is usually discussed in relation to the harmonic imaging method (HIM), which is a software algorithm used to obtain high-resolution images [57]. The harmonic distortion components of the echo signals generated by a transducer can be minimized in the signal-processing step. Some commercial power amplifiers provide harmonic distortion parameters in the datasheet so that researchers can estimate the extent to which the signal distortion of the power amplifier can affect the signal quality of the transducer. 

Efficiency is an important parameter, particularly in wireless ultrasound systems [58,59,60]. It indicates how long the battery in the system can provide DC voltages. Efficiency can be regarded as an index showing how efficiently the energy can generate the output power to trigger the transducer. The drain efficiency or power-added power efficiency (PAE) parameters are also used in power amplifiers. The drain and power efficiency values are similar if the output power is much larger than the input power [61]. The power amplifier with high efficiency normally generates high signal distortions, so it could produce relatively high harmonic distortions from the transducers, which lowers the image resolution of the ultrasound system [62]. Therefore, we need to consider efficiency and signal distortion together. For a power amplifier with high efficiency, we need to be concerned about some effects, especially when choosing the transistors, which are one of the main components of the power amplifiers. For the transistors, we need to consider the voltage, current, power, and frequency limitation, even with appropriate parasitic capacitance values. For transducer excitation, high voltage or high power generation is required. For example, the transistor with high voltage or high power typically has high parasitic capacitance values, which could affect signal distortions [56]. Therefore, the proper transistor selection for the power amplifier with high efficiency is very challenging work because the transistor must work proper power and frequency ranges during the operation, even though we want to design a power amplifier with high gain and high efficiency.

The linearity indicates the extent to which the input voltage of the power amplifier is increased [63,64]. The power amplifier contains input or output intermodulation points (IIP_3_ or OIP_3_). A higher IIP_3_ or OIP_3_ indicates that the input voltage, current, or power can be amplified to generate an output voltage, current, or power with little signal distortion up to that point (IIP_3_ or OIP_3_) [65,66]. Linearity is also related to the signal or harmonic distortion. For example, nonlinear power amplifiers usually have high efficiency but low linearity. 

**Figure 2:**
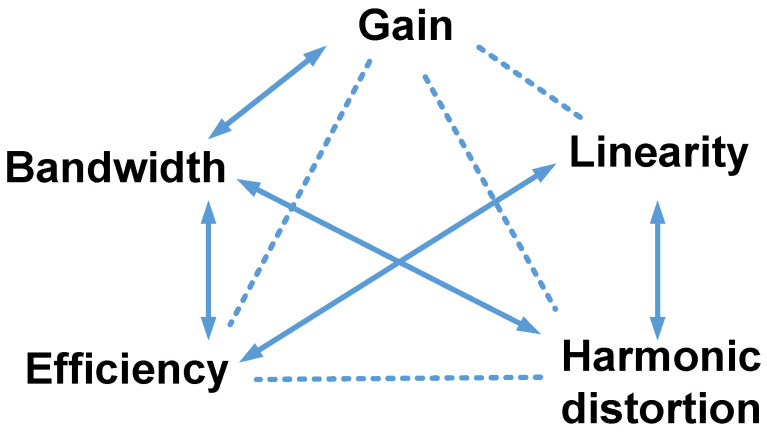
Design parameters of power amplifiers for ultrasound applications. The arrows represent inversely proportional relationships, and the dotted lines indicate other relationships.

Figure 2 shows a symbol diagram of the design parameters to show the specific relationships because some design parameters have trade-off relationships. Basic information was obtained from the integrated circuit and power amplifier textbooks. For example, a higher gain for a power amplifier can lower the bandwidth [46]. Power amplifiers with a wide bandwidth may have low efficiency [67]. In Figure 2, the relationships between the parameters indicate that there are challenges in obtaining a high-performance power amplifier. Therefore, circuit design engineers must have the experience and intuition needed to obtain the proper performance from a power amplifier.

## 3. Classifications of Power Amplifiers

This section describes the fundamental concept of each class of power amplifiers and the specifications and design methodologies of the previously reported power amplifiers used for ultrasound applications. The specific design methodologies for these power amplifiers provide guidelines for ultrasound research. This section focuses on a practical approach to power amplifier design, which can be used when selecting components to attain the best power amplifier performance. Although other power amplifiers have been developed, Class-A to Class-F power amplifiers are considered here because these have been widely utilized for WiFi, wireless, ultra-wideband, instrumentation, aerospace, and ultrasound applications [68,69].

### 3.1. Class-A Power Amplifier

A Class-A power amplifier is the most linear power amplifier with the highest DC power consumption among all the power amplifier classes because the output voltage and current are assumed to be in the same phase [70]. Therefore, the thermal heat generated by the power amplifier transistor can directly affect the battery of a wireless ultrasound system. There may be low signal distortions in the amplified output signals. In addition, a Class-A power amplifier is preferable for use with the harmonic imaging method, which requires low harmonic distortion because of its high linearity [70].

**Figure 3:**
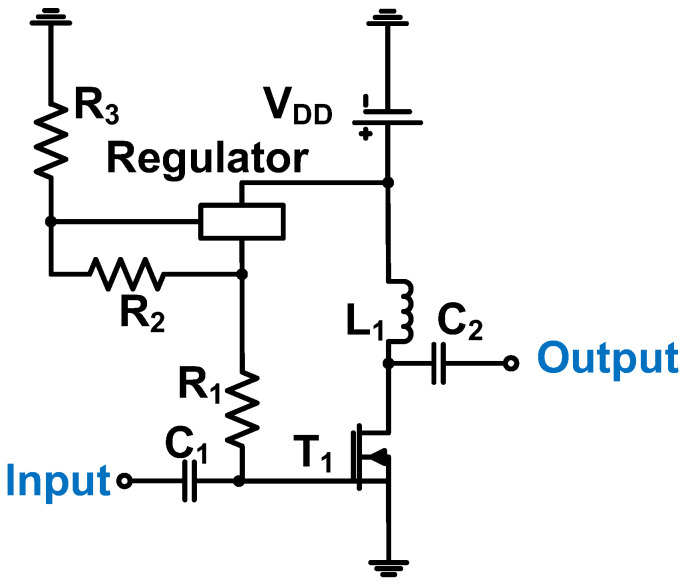
Class-A power amplifier schematic diagram for piezoelectric transducer. Adapted from Choi, H et al. Ref. [71] with permission under the terms of the CC BY 4.0 License, Copyright 2017 MDPI AG.

As shown in Figure 3, the common-source amplifier scheme was used with a typical voltage divider using resistors (R_2_, Regulator, and R_3_) with input and output coupling capacitors (C_1_ and C_2_) to remove the DC voltages. A choke inductor (L_1_) was used to apply the maximum DC voltage to the drain of the transistor [70]. Capacitors were used to filter out the high-frequency noise from the DC power supply [70]. The operating frequency of the Class-A power amplifier was 10 MHz, the output power was 14.21 dB_m_, and the voltage gain was 15.6 dB.

**Figure 4:**
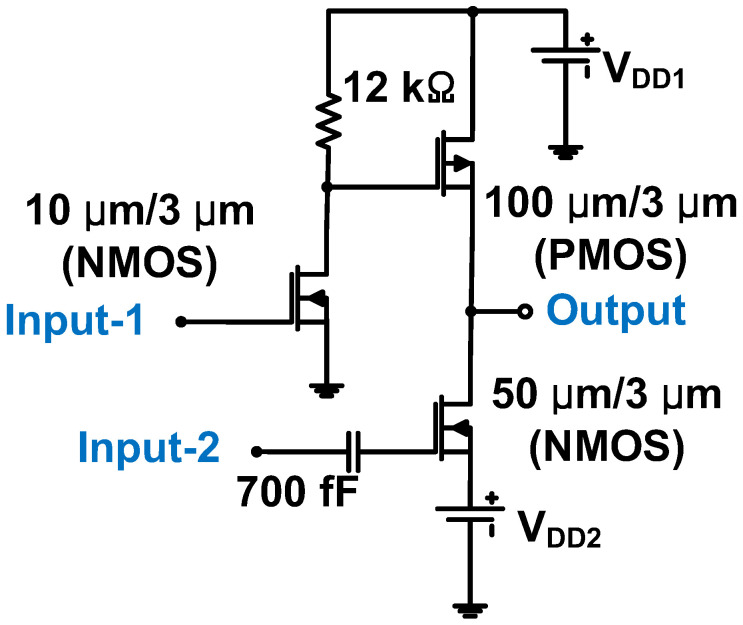
Class-A power amplifier for 2D CMUT array [73].

As shown in Figure 4, a Class-A transmitter amplifier with an output voltage of 15 V was designed for a 2 MHz 2D CMUT array. Bias voltages were provided from a +45 V DC supply to the gate and drain of the n-channel MOS (NMOS = 10 μm/3 μm and 50 μm/3 μm) and p-channel MOS (PMOS = 100 μm/3 μm) transistors. The resistor (12 kΩ) reduced the DC supply such that the PMOS transistor functioned as a diode-connected load to increase the gain [72]. 

### 3.2. Class-B/AB Power Amplifier

In ultrasound system papers, Class-B/AB power amplifiers are called pulsers or pulse generators [62]. Most of the power amplifiers currently used for ultrasound applications are this type because positive and negative power supplies can provide wider ranges of DC voltages than a single-side power supply. The maximum reachable voltage gain is theoretically double that of a Class-A power amplifier with minimum signal distortion [74]. However, the digital-to-analog converter (DAC) and digital control logic circuits must generate two different positive and negative inputs to the power amplifiers [75]. Therefore, accurate control of the input signals and positive/negative DC voltages is required to maintain the stability of the output signals. Impedance matching and filtering must be carefully performed to reduce unwanted high-frequency noise signals [76]. Therefore, ultrasound circuit designers must check the frequency response graph using an oscilloscope during system development.

**Figure 5:**
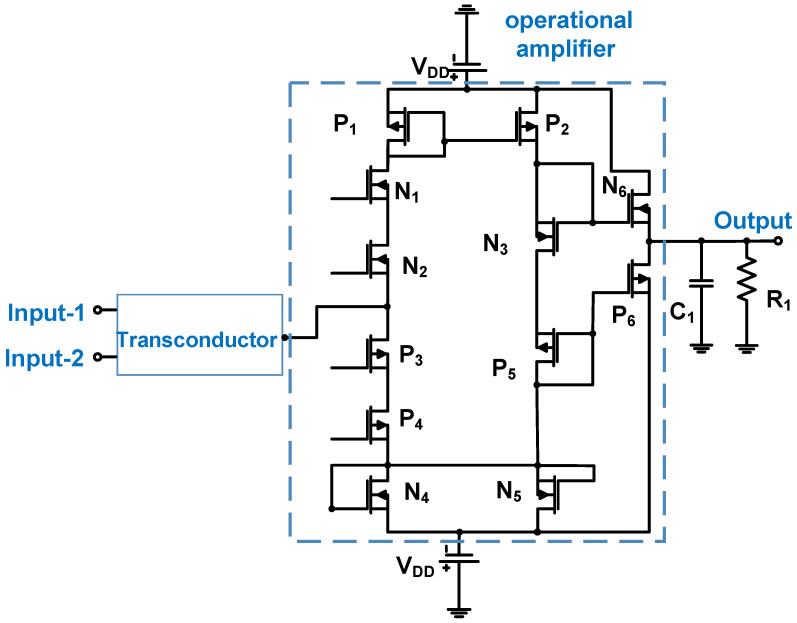
Class-B power amplifier schematic diagram (the bias voltage connection is not shown to simplify the analysis) [79].

The total harmonic distortion of the power amplifier can be controlled using a low-pass or band-pass filter architecture [77]. As shown in Figure 5, the Class-B power amplifier uses a transconductor, differential cascode transimpedance amplifier, and buffer architecture. The transconductor is used to change the voltage input into the current input of the differential cascade transimpedance amplifier [77]. In addition, the transconductor can provide a high impedance because the ideal input and output impedances of the transimpedance amplifier are infinite and zero, respectively [78]. 

In Figure 5, Bianchi et al. used a transconductor, operational amplifier, and buffer in a power amplifier design [79]. Low- and high-voltage supplies were applied to the transconductor, and bias voltages were applied to the differential operational amplifier. The use of two separate supply voltages (V_DD_) assisted in removing noise from the regulator circuit [79]. The NMOSs (N_1_–N_6_) and PMOSs (P_1_–P_6_) were used by the differential cascade transimpedance amplifier to provide the power amplifier gain. The buffer amplifier (N_6_ and P_6_) was the source follower, which provided a unity gain to reduce the output impedance [78]. The buffer amplifier was used to change the current input into a voltage output with low impedance to match the ultrasound transducer (C_1_ = 150 pF and R_1_ = 100 Ω) [79]. It produced an output voltage of 90 V_p-p_, a voltage gain of 40.9 dB, a bandwidth of 6.5 MHz, and a second harmonic distortion of approximately −35 dB.

**Figure 6:**
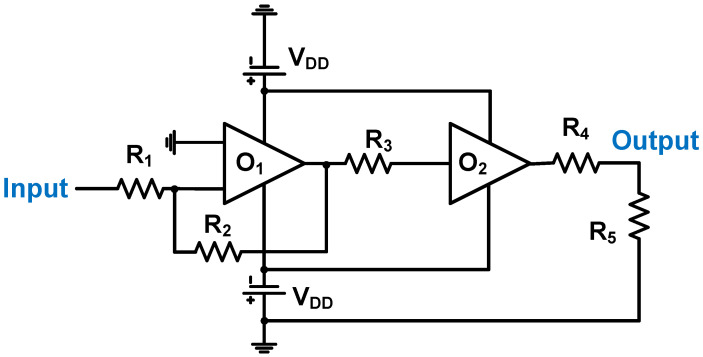
Schematic diagram of Class-AB power amplifier for power piezoelectric transducer Apdated from L. Capineri et al. Ref. [80]. Copyright 2014, AIP Publishing.

As shown in Figure 6, a Class-AB power amplifier uses an operational amplifier (O_1_) with a resistive feedback loop and buffer (O_2_) architecture designed for a 10 MHz ultrasonic transducer [80]. The bandwidth of the power amplifier could be extended to 15 MHz using a resistive feedback loop [80]. A feedback resistor (R_2_ = 1 kΩ) was used to increase the bandwidth, thereby sacrificing the maximum voltage gain, and a series resistor (R_3_ = 215 Ω) was used to smoothly pass the output signals, even though the amplified output voltage was suppressed [80]. The gain of the power amplifier was dependent on two resistors (R_1_ = 100 Ω and R_2_ = 1 kΩ). The measured output voltage was 27.25 V, the output power was 3.09 W, and the efficiency was 5.66% at 10 MHz.

**Figure 7:**
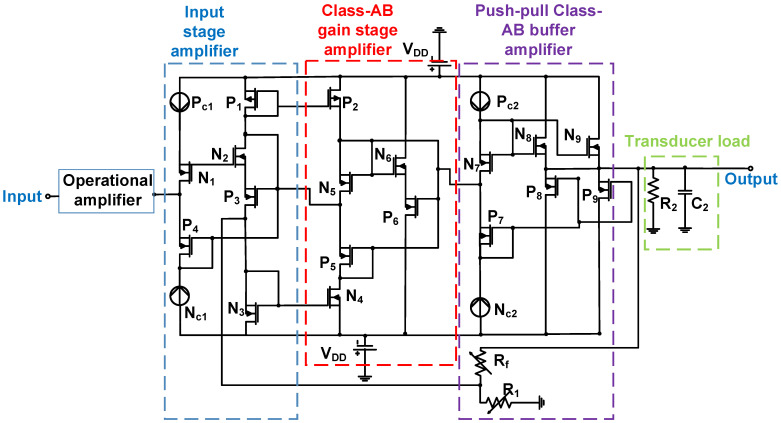
Current feedback linear power amplifier schematic diagram [81].

As shown in Figure 7, an operational amplifier with resistive feedback resistors increased the low-voltage input signal in the voltage follower (the input-stage amplifier). The generated output voltage from the voltage follower was fed into the Class-AB gain-stage amplifier and amplified in the push–pull Class-AB buffer amplifier. Resistor R_2_ and capacitor C_2_ were the parasitic resistor and capacitor in the equivalent circuit of the transducer load, respectively [81]. The amplified high-voltage signals from the Class-AB gain-stage amplifier were passed to the push–pull Class-AB buffer amplifier with variable resistors (R_1_ and R_f_), which were dependent on the gain of the final-stage buffer amplifier [81]. The output buffer amplifier was used to provide a low impedance and isolate the performance between the push–pull Class-AB power amplifier and transducer load so that the performance of the Class-AB power amplifier could be controlled during operation [81]. For the designed Class-AB power amplifier, the measured output voltage was 180 V_p-p_, the measured total harmonic distortion (THD) was −48 dB, and the −3 dB bandwidth was 8.6 MHz.

**Figure 8:**
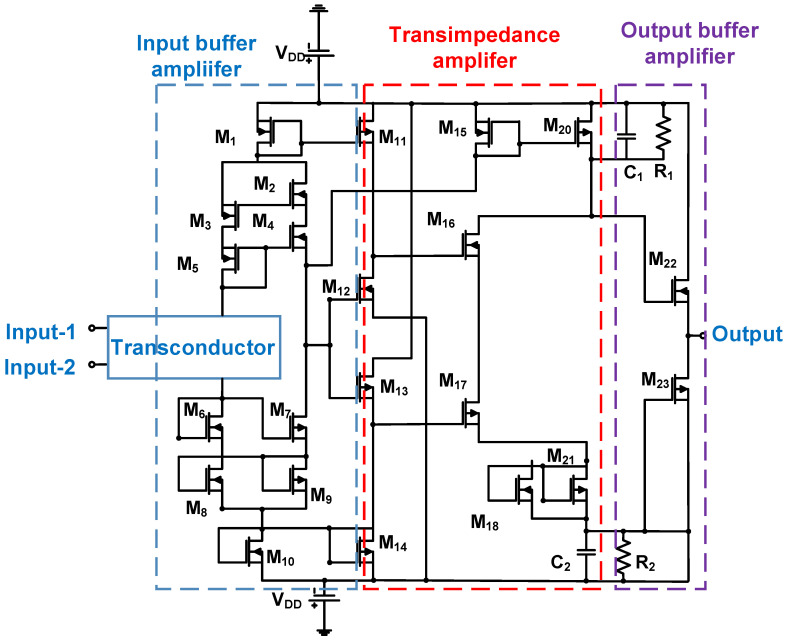
Class-AB power amplifier schematic diagram [84].

As shown in Figure 8, a Class-AB power amplifier is composed of a transconductor, an input buffer amplifier, a transimpedance amplifier, an output buffer amplifier, and a current multiplier. The transconductor is used to convert the current into a voltage signal. The input buffer amplifier is a low-voltage amplifier that provides relatively high-amplitude input signals. The transimpedance amplifier is a two-stage operational amplifier with a cascade architecture that increases the transconductance values of the transistors, thereby improving the total gain of the power amplifier. The current mirror uses the Wilson topology to increase the output impedance [82]. The output buffer amplifier is a one-stage source follower type that lowers the output impedances [83]. Current multipliers (M_22_ and M_23_) were used to increase the final output current [84]. The measured output voltage was 180 V, −3 dB bandwidth was 20 MHz, and second harmonic distortion was −48 dB.

**Figure 9:**
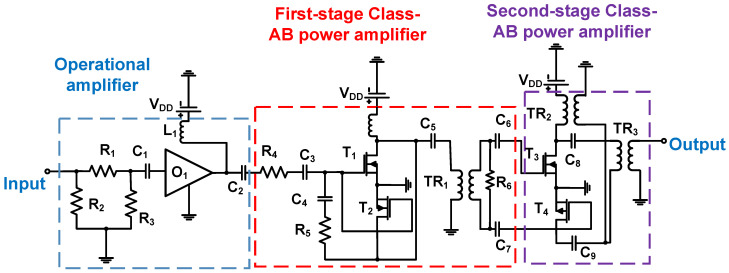
Class-AB power amplifier for neuro-stimulation applications (the bias voltage connection is not shown to simplify the analysis) [85].

In a Class-AB power amplifier (Figure 9), the operational amplifier (O_1_) is used to increase the input voltage generated by the DAC, and the increased output is fed into the first-stage Class-AB power amplifier with DMOSs T_1_ and T_2_, and the second-stage Class-AB power amplifier with DMOSs T_3_ and T_4_ [85]. The first transformer (TR_1_) is used to split one signal into two signals (positive and negative), and the second transformer (TR_2_) is used to combine the two signals into one signal [85]. Several capacitors (C_1_, C_2_, C_4_, C_5_, C_6_, C_7_, and C_8_) are used to remove the DC voltage and pass the AC signals. An impedance-matching circuit using transformers is also used to reduce the output power reflection from the ultrasound transducer [85]. The maximum operating frequency, output voltage amplitude, and gain were 5 MHz, 48 V_p-p_, and 40 dB, respectively. 

### 3.3. Class-C Power Amplifier

Class-C power amplifiers have been used in mobile communication applications [86]. A Class-C power amplifier theoretically has a low power consumption compared with Class-A and Class-B/AB power amplifiers, but it has high signal distortions [87]. For Class-C power amplifiers, the voltage and current are out of phase with each other to achieve low DC power consumption [87]. To reduce the signal distortions of Class-C power amplifiers, delicate biasing circuits must be utilized to provide a stable DC supply voltage [88].

**Figure 10:**
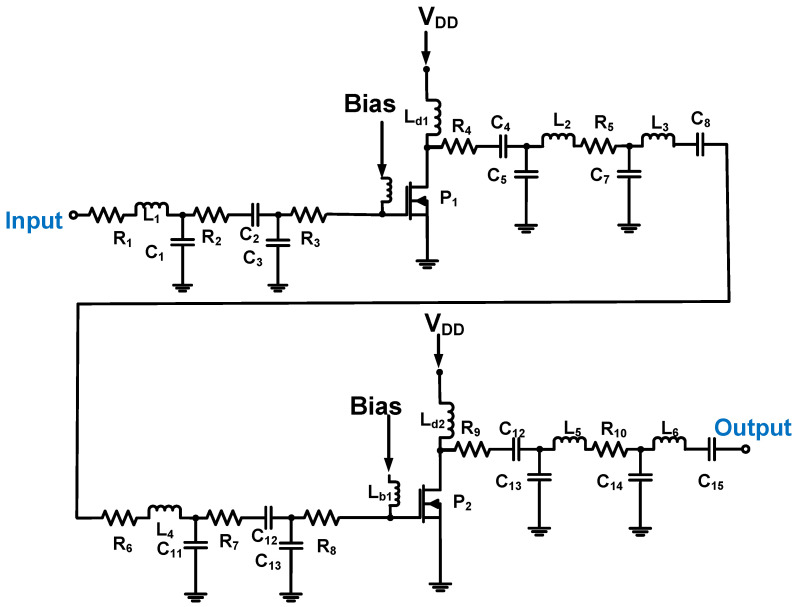
Class-C power amplifier schematic diagram (the bias voltage connection is not shown to simplify the analysis). Adapted from Choi, H. Ref. [89] with permission under the terms of the CC BY 4.0 License, Copyright 2019, MDPI AG.

As shown in Figure 10, a two-stage Class-C power amplifier was designed [89]. To reduce the power consumption, the current and voltage requirements at different conduction angles were satisfied. The input and output matching circuits use resistors, capacitors, and inductors to achieve 50 Ω for the power amplifier, and the series resistors (R_3_ and R_4_) in the input and output matching circuits are used to pass signals with fewer distortions [89]. For the designed Class-C power amplifier, the measured voltage gain was 17.14 dB, the center frequency was 25 MHz, and the DC power consumption was 0.975 W.

### 3.4. Class-D/DE Power Amplifier

The Class-D power amplifier can produce a high-voltage output, but generates high-voltage signal distortion because the input signals of the power amplifier are modulated pulse signals with user-defined functions [90]. Therefore, various digital combinational circuits can easily generate modulated input signals such as square, triangular, and Gaussian pulses [90]. To reduce high-voltage signal distortions, high-order low-pass digital filters and complex combinational logic circuits are required before producing modulated input signals, although they cannot completely remove signal distortions [91]. This requirement can increase the fabrication cost of Class-D power amplifiers. Therefore, the high THD of the output signals generated by Class-D power amplifiers must be considered in ultrasound applications. The theoretical efficiency of a Class-D power amplifier is close to 90% at the resonant frequency; therefore, it is relatively easy to handle heat sink problems produced by high-voltage or high-power signals for ultrasound applications [92]. Therefore, Class-D power amplifiers are suitable for high-power piezoelectric loads.

**Figure 11:**
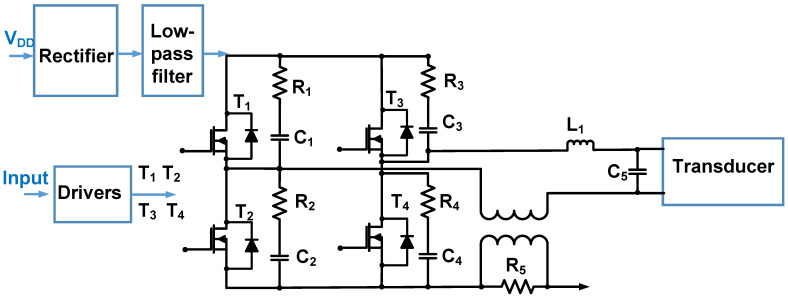
Class-D power amplifier schematic for power piezoelectric load [93].

Figure 11 shows a schematic of the Class-D power amplifier used for the power piezoelectric load. Positive and negative pulse-modulated signals between 10 kHz and 100 kHz were produced by the drivers [93]. A rectifier and a low-pass filter were used to provide the supply voltage. Four power MOSFETs (T_1_, T_2_, T_3_, and T_4_) were used as switches [93]. If T_1_ and T_4_ were turned on, the output voltage was close to that of the positive supply. If T_2_ and T_3_ were turned on, the output voltage was close to that of the negative supply. To reduce the high-frequency noise, an additional capacitor and resistor were used next to the MOSFETs [93]. A low-pass Butterworth filter (C_5_ and L_1_) was used because it can provide a relatively flat magnitude during low-frequency operation [93]. The Class-D power amplifier operated with an output power of 2000 W for the resistor load and 1500 W for the reactive load.

**Figure 12:**
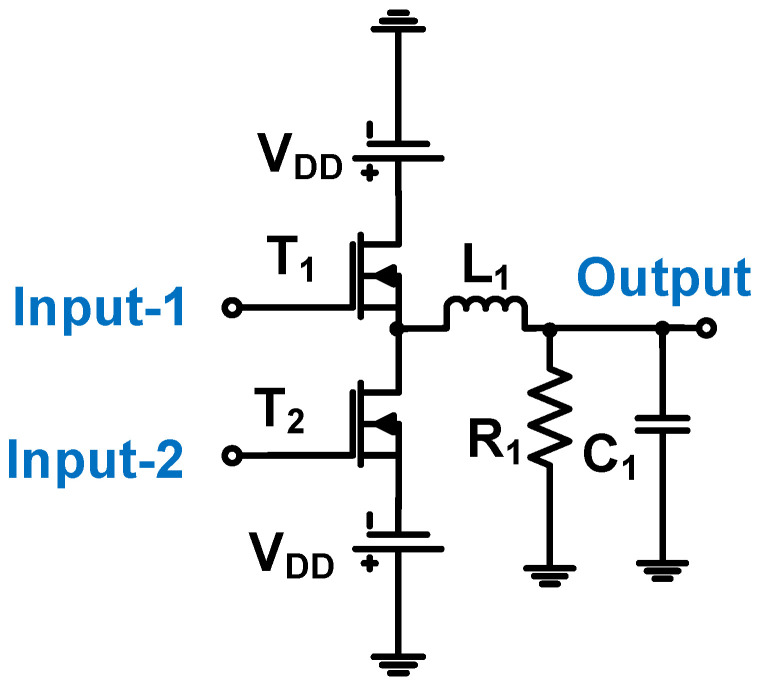
Schematic of Class-D power amplifier for dielectric elastomer transducer [94].

In Figure 12, a zero-voltage switching mechanism is used such that one MOSFET is turned on and another MOSFET is turned off during the 50% duty cycle [94]. The turn-on/off time is dependent on the values of the inductor (L_1_), transistor parasitic drain-source parasitic capacitance (C_DS_), and capacitor (C_1_) [94]. An output gain of 43.5 dB, a resonant frequency of 0.1 kHz, and an efficiency of 42% was obtained from the Class-D power amplifier.

**Figure 13:**
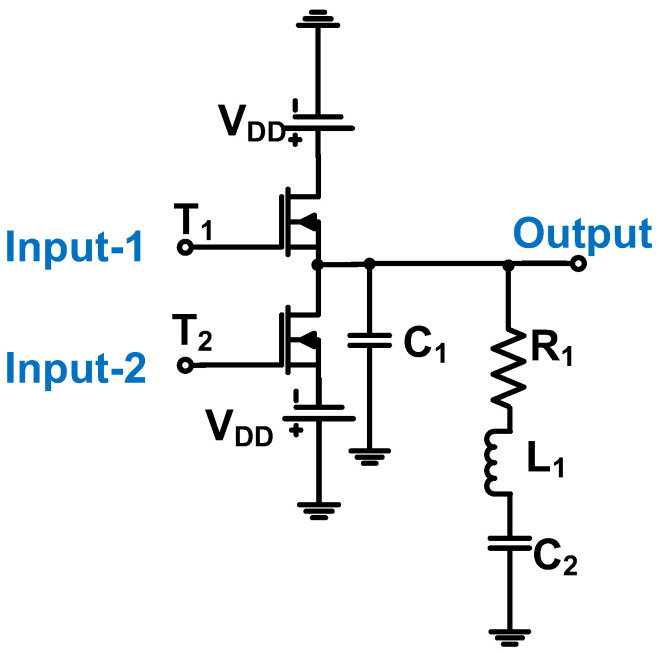
Schematic of Class-DE power amplifier used for HIFU therapy [95].

Figure 13 shows a schematic of a Class-DE power amplifier that is compatible with an MRI machine. Zero-voltage and zero-current switching mechanisms were used to determine the operating frequency of the power amplifier [95]. Therefore, a transducer equivalent circuit model composed of a resistor (R_1_), an inductor (L_1_), and a capacitor (C_2_) was defined [95]. Two MOSFETs (T_1_ and T_2_) were used as switches. Capacitor C_1_ represents the combined capacitance of the transducer capacitor, output capacitances of the two MOSFETs, and external capacitances [95]. The operating frequency of the Class-DE power amplifier was 1010 kHz. The third harmonic distortion component was −16.4 dB, and the output power was 0.83 W.

### 3.5. Class-E Power Amplifier

In a previous study, a Class-E power amplifier was also called a Class-E inverter because of the switching characteristics of the MOSFET [96]. The values of several components in the output port can be calculated using the theoretical parameter equations provided by the typical Class-E power amplifier theory [96]. However, the specific frequency of the filtering function needs to be adjusted using the theoretical parameters to obtain optimal performance [96]. In previous studies, several Class-E power amplifiers have been used for ultrasound applications, as described here. 

**Figure 14:**
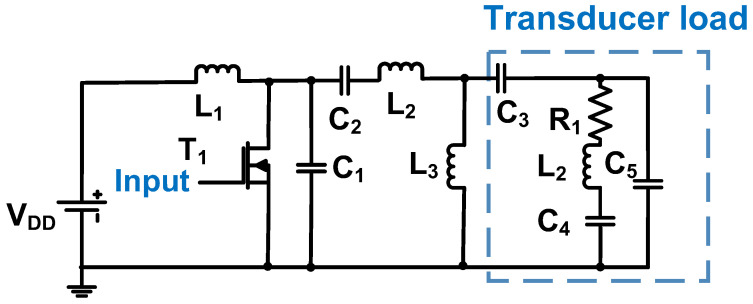
Class-E power amplifier schematic diagram. Adapted with permission from Ref. [96]. Copyright 2017, Elsevier.

Figure 14 shows the Class-E power amplifier with the impedance of the ultrasonic transducers (R_1_, L_2_, C_4_, and C_5_) in the equivalent circuit model. Capacitor C_3_ was obtained using the impedance of the ultrasonic transducer and the optimal frequency based on the resonance network theory [96]. The two inductors (L_1_ and L_2_) were obtained using the impedance transformation theory [97]. The optimum frequency was 41.27 kHz, the output voltage was 58.76 V, and the output power was 0.17 W.

**Figure 15:**
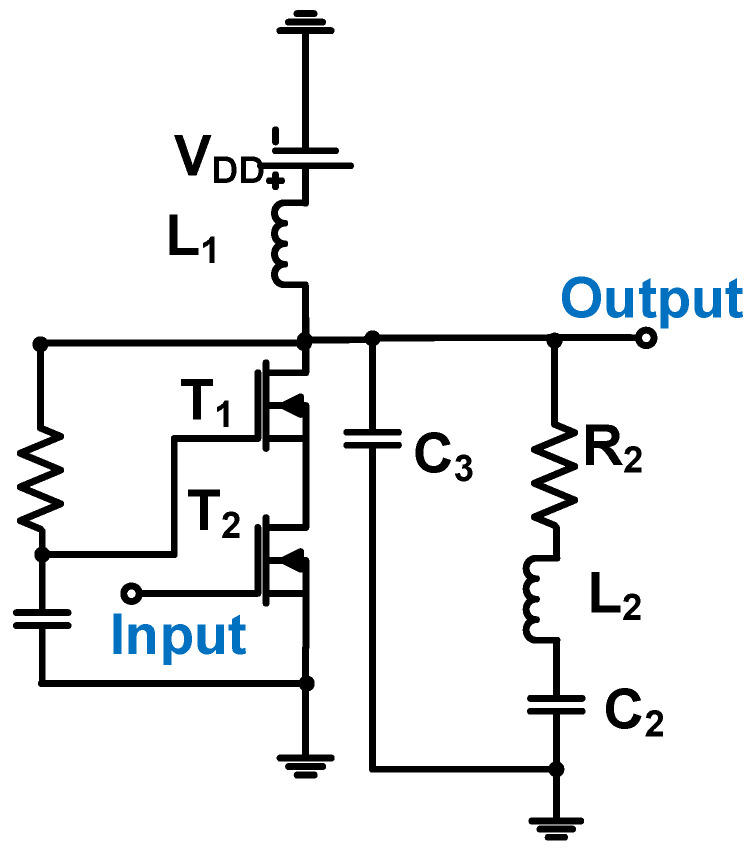
Self-biased cascade Class-E power amplifier schematic diagram. Adapted with permission from Ref. [98]. Copyright 2020, Elsevier.

Figure 15 shows a self-biased Class-E power amplifier for the 32 MHz CMUT used in HIFU applications. The CMUT impedance (C_3_, R_2_, L_2_, and C_2_) is schematically shown in the equivalent circuit model [98]. A resistor and capacitor (R_1_ and C_1_) with a low-pass filter were used to reduce the output voltage stress to the MOSFETs (T_1_ and T_2_), thus reducing the overshoot of the output voltages or currents [98]. However, this could affect the operating frequency of the power amplifier. Inductor L_1_ and resistor R_1_ could reduce the DC voltage from the supply (V_DD_) to provide a reduced DC voltage to the gates of the transistors (T_1_ and T_2_) without a separate biasing circuit [98]. The Class-E power amplifier produced an output voltage of 36.5 V and a center frequency of 32 MHz.

**Figure 16:**
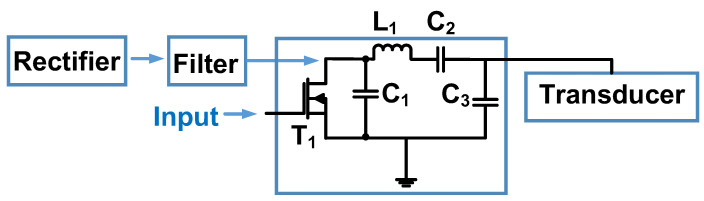
Schematic diagram of Class-E resonant inverter for a piezoelectric transducer [99].

Figure 16 shows a Class-E resonant inverter based on a zero-voltage switching mechanism for a piezoelectric transducer. The switch can be turned on if the gate–source voltage of the MOSFET (T_1_) is a high-voltage pulse signal, and turned off if the gate–source voltage of the MOSFET (T_1_) is a low-voltage or negative high-voltage pulse signal [99]. The inductor and capacitor components (L_1_, C_1_, and C_2_) can be calculated based on the equations for a theoretical Class-E power amplifier [99]. The maximum output voltage and current were 112 V and 0.364 A, respectively, and were delivered at a frequency of 28.11 kHz.

### 3.6. Class-F Power Amplifier

**Figure 17:**
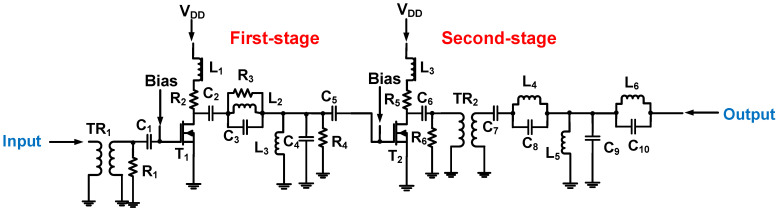
Schematic diagram of the Class-F power amplifier (the bias voltage connection is not shown to simplify the analysis). Adapted from Kim, K et al. Ref. [100] with permission under the terms of the CC BY 4.0 License, Copyright 2021, Plos One.

Compared to Class-E power amplifiers, Class-F power amplifiers are useful for filtering out the second harmonic distortion generated by the power amplifier itself [75]. A Class-F power amplifier also has a relatively high power efficiency. As shown in Figure 17, a two-stage Class-F power amplifier was designed to resonate with the second harmonic component, thus lowering the second harmonic distortion [100]. Harmonic signals were reduced using a resonant filter network in the first- and second-stage power amplifiers [100]. The generated output power was 33.5 dB_m_, the gain was 23.5 dB, the center frequency was 25 MHz, the power added efficiency was 78.8%, and the total harmonic distortion was 5%.

### 3.7. Power Amplifier Classification

Table 1 summarizes the power amplifiers currently used for ultrasound applications, from Class-A to Class-F power amplifiers, along with their design parameters. Table 1 does not list all the power amplifiers developed for ultrasound applications. However, the summarized parameters of the listed power amplifiers could assist in understanding the power amplifier trend in ultrasound research. The frequency is the operating or center frequency, and the bandwidth is the −3 dB bandwidth. The harmonics represent the second harmonic distortion (HD2) or THD, and the efficiency is the drain efficiency or PAE.

## 4. Discussion

This review summarizes previous power amplifier research for ultrasound applications to guide design and analysis. A variety of class mode power amplifiers have been discussed for ultrasound applications, along with transistor selection and design parameters.

First, selecting an appropriate transistor is important. The maximum gate–source and drain–source voltages of the transistor could be a concern because the desired output voltage of the power amplifier is related to these voltage ranges [40]. The maximum drain current of the transistor must be considered because of the potential for transistor failure owing to the maximum DC power consumption [49]. The parasitic gate–source, gate–drain, and drain–source capacitances must be considered because of the maximum operating frequency of the power amplifier itself [52]. After the careful selection of the transistor, design parameters such as the gain, bandwidth, harmonic distortion, linearity, and efficiency need to be considered. In general, a high-gain power amplifier is desirable because a low-sensitivity ultrasonic transducer requires high-voltage or high-current signals to be triggered [101]. The power amplifier bandwidth must be higher than that of the transducer [24]. The efficiency is related to the battery of a wireless ultrasound system; therefore, high efficiency is desirable for efficient power consumption. A high linearity is desirable because the output voltage of the power amplifier can be amplified with less signal distortion [64].

A Class-A power amplifier can produce a highly linear output voltage, current, or power signal. Unfortunately, it generates the highest amount of thermal energy among all the class mode power amplifiers [70]. Therefore, a Class-A power amplifier is more suitable for imaging applications rather than wireless ultrasound systems. The Class-B/AB power amplifier has been the most widely used power amplifier type in previous ultrasound studies. A Class-B/AB power amplifier is called a pulser or pulse generator [62]. Using positive and negative power supplies for the power amplifier can help widen the DC voltage range. However, accurate DACs and digital control logic circuits are required for a stable power amplifier design [77]. Delicate impedance matching and filter tuning processes are also required to minimize unwanted high-frequency noise [75]. A Class-C power amplifier has a low DC power consumption with high signal distortion. To reduce signal distortion, delicate biasing circuits must be used to stabilize the supply voltage [86]. A Class-D/DE power amplifier can provide a high power efficiency but generates high signal distortions. A low-pass filter is typically used to reduce signal distortion, although high-frequency harmonic signals cannot be eliminated [75]. However, the modulated signals can be used in digital combinational logic circuits [90]. The output components of a Class-E power amplifier were specified using device parameter equations. To obtain the optimal operating frequency, trial-and-error methods using various discrete components were employed based on the equivalent circuit model of an ultrasonic transducer [96]. A Class-F power amplifier is useful for filtering the second harmonic distortion of a power amplifier [75]. Therefore, echo signals with high second harmonics generated by an ultrasound transducer could be beneficial. In addition, high efficiency can be achieved; therefore, a Class-F power amplifier scheme could be useful for wireless ultrasound systems. 

Table 2 summarizes the contributions and limitations of the previously designed power amplifiers used for ultrasound research to assist in understanding the types of power amplifiers that are useful for specific ultrasound applications. The contributions and limitations of these power amplifiers listed in Table 2 provide guidelines for power amplifier designers and ultrasound researchers.

## 5. Conclusions

There have been many review papers on the power amplifiers used in communication system applications. However, no review papers have provided design guidelines or design parameter specifications for the power amplifiers used in ultrasound applications. Therefore, this review described the types of ultrasound applications in which power amplifiers are properly used and the types of design parameters that are commonly utilized for specific ultrasound applications. 

Currently, ultrasound systems are becoming more complex because of the different combinations of MRI, optical imaging, and PET being utilized. They are also becoming smaller because of the requirements for wireless ultrasound machines. Therefore, this review could be helpful for design engineers and ultrasound researchers. Several design guidelines exist for the power amplifiers used in ultrasound applications. For design engineers, this paper provides the detailed specifications and design parameters of power amplifiers. This review could also be helpful for ultrasound researchers who are not familiar with electronics because it provides guidelines on which types of power amplifiers need to be purchased for ultrasound research. There are several parameters for power amplifiers, including gain, bandwidth, harmonic distortion, linearity, and efficiency. Unfortunately, all the parameters have trade-off relationships because of the use of nonlinear electronic components such as transistors, capacitors, and inductors. For each ultrasound application, certain types of power amplifiers could help achieve the proper performance for ultrasound research.

## Figures and Tables

**Table 1:** Summary of the power amplifiers currently used for ultrasound research.

Paper	Class Mode	Operating or Center Frequency	Output Power	Output Voltage	−3 dB Bandwidth	Gain	Harmonic Distortion or THD	Efficiency or PAE	Application
[71]	Class-A	10 MHz	14.21 dB_m_	-	-	15.6 dB	-	-	Piezoelectric Transducer
[73]	Class-A	2 MHz	-	15 V	-	-	-	-	CMUT
[79]	Class-B	-	-	-	6.5 MHz	40.9 dB	<−35 dB (HD2)	-	Ultrasonic Transducer
[80]	Class-AB	-	3.09 W	27.25 V	15 MHz	-	-	5.66%	Ultrasonic Transducer
[81]	Class-AB	-	-	180 V	8.6 MHz	-	−48 dB	-	Piezoelectric Transducer
[84]	Class-AB	-	-	180 V	22 MHz	-	−48 dB	-	Medical Echography
[85]	Class-AB	5 MHz	-	48 V	-	40 dB	-	-	Neuromodulation
[89]	Class-C	25 MHz	-	-	-	17.14 dB	-	-	Piezoelectric Transducer
[93]	Class-D	100 kHz	2000 W	-	-	-	-	-	Power Piezoelectric Load
[94]	Class-D	0.1 kHz		-	-	43.5 dB	-	42%	Dielectric Elastomer Transducer
[95]	Class-DE	1010 kHz	0.83 W	-	-	-	−16.4 dB (HD2)	-	HIFU Therapy
[96]	Class-E	40.07 kHz	0.219 W	-	-	-	-	-	Langevin Piezoelectric Transducer
[98]	Class-E	32 MHz	-	36.5 V	-	-	-	-	CMUT
[99]	Class-E	28.11 kHz	-	112 V	-	-	-	-	Piezoelectric Transducer
[100]	Class-F	25 MHz	33.5 dB_m_	-	-	23.5 dB	5.0%	78.8%	Piezoelectric Transducer

**Table 2:** Contributions and limitations of power amplifiers for specific ultrasound applications.

Class Mode	Contribution	Limitation
Class-A	Highly linear characteristics and thus preferable for ultrasound imaging applications.	Because it has low efficiency, it could not be recommended for a wireless ultrasound machine.
Class-AB/B	The positive and negative DC supply makes a high gain achievable.	A combinational logic circuit and stable DAC circuit are necessary.
Class-C	Low DC power consumption can be obtained.	A delicate biasing circuit topology is necessary.
Class-D/DE	High power efficiency can be obtained. The modulated pulse signals can be generated by a user-defined function.	Because of the high signal distortion, high-order low-pass filters and complex combinational logic circuits are required.
Class-E	Output matching circuit equations can provide the proper component values for the power amplifier.	A fine-tuning method is needed to achieve an appropriate current and voltage phase condition.
Class-F	High efficiency can be obtained.	A delicate harmonic distortion filter is required.

## Data Availability

Data are contained within the article.

## Abbreviations

DC	Direct current
AC	Alternating current
DAC	Digital-to-analog converter
MRI	Magnetic resonance imaging
PET	Positron emission tomography
HIFU	High-intensity focused ultrasound
CMUT	Capacitive micromachined ultrasonic transducer
HIM	Harmonic imaging method
IIP_3_	Third input intercept point
OIP_3_	Third output intercept point
PAE	Power added power efficiency
MOSFET	Metal-oxide-semiconductor field-effect transistor
HD2	Second harmonic distortion
THD	Total harmonic distortion
LDMOS	Lateral diffusion metal-oxide semiconductor
DMOS	Double-diffused metal-oxide semiconductor
NMOS	N-channel MOS
PMOS	P-channel MOS
